# Schwann Cell‐Specific ATG7‐Dependent Autophagy Protects Myelin in a Mouse Model of Krabbe Disease

**DOI:** 10.1002/glia.70244

**Published:** 2026-10-11

**Authors:** Narayan Dhimal, Aditya Sidharth, Jiajun Zhang, Trina Rudra, Edward Hurley, Pablo M. Paez, Fraser J. Sim, Daesung Shin, M. Laura Feltri

**Affiliations:** ^1^ Institute of Myelin and Glia Exploration Jacobs School of Medicine and Biomedical Sciences, University at Buffalo, State University of New York Buffalo New York USA; ^2^ Neuroscience Program Jacobs School of Medicine and Biomedical Sciences, University at Buffalo, State University of New York Buffalo New York USA; ^3^ Department of Biotechnical and Clinical Laboratory Sciences Jacobs School of Medicine and Biomedical Sciences, University at Buffalo, State University of New York Buffalo New York USA; ^4^ Department of Pharmacology and Toxicology Jacobs School of Medicine and Biomedical Sciences, University at Buffalo, State University of New York Buffalo New York USA; ^5^ Department of Biochemistry and Neurology Jacobs School of Medicine and Biomedical Sciences, University at Buffalo, State University of New York Buffalo New York USA

## Abstract

Krabbe disease (KD) is a lysosomal storage disorder characterized by severe peripheral demyelination caused by mutations in galactosylceramidase (GALC), leading to the accumulation of galactosylceramide and psychosine. Although psychosine toxicity is central to KD pathology, additional mechanisms likely contribute to myelin dysfunction. Because Schwann cells (SCs) play a critical role in myelin maintenance, and GALC deficiency disrupts lysosomal function, we hypothesized that autophagy is altered in SCs during KD progression. Using a SC‐specific *Galc* conditional knockout (*Galc* SC cKO) mouse model, we identified early changes in autophagy‐related protein levels and increased autophagosome abundance during the pre‐symptomatic phase, prior to detectable myelin abnormalities. To assess whether autophagy contributes to disease progression, we selectively deleted *Atg7*, an essential regulator of canonical autophagy, in SCs. *Atg7* deletion alone did not affect myelin or behavior, but its loss in *Galc* SC cKO mice markedly worsened disease severity. *Atg7‐Galc* SC cKO mice exhibited significantly thinner myelin and pronounced deficits in motor performance and nerve conduction, accompanied by altered steady‐state levels of autophagy‐associated proteins and functional decline. Notably, *Atg7* deletion in *Galc* SC cKO mice did not further increase macrophage abundance, suggesting that the worsened myelin phenotype occurs without additional macrophage recruitment at this stage. Together, these findings demonstrate that autophagy‐associated changes occur early in KD and ATG7‐dependent pathways play a protective role during disease progression. Modulating ATG7‐dependent pathways may therefore warrant further investigation as a strategy to support myelin maintenance in KD and related lysosomal disorders.

## Graphical abstract

Krabbe disease (KD) is caused by mutations in *GALC* and deregulated lysosomal function in Schwann cells (SCs). Using a SC‐specific *Galc*‐cKO mouse, we identified altered autophagy in SCs during KD progression. Further ablation of *Atg7* in SCs of KD worsened disease severity.

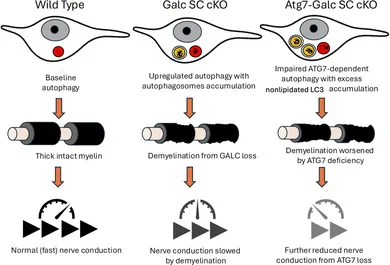

## 1 Introduction

Krabbe disease (KD), also known as Globoid Cell Leukodystrophy, is a rare and devastating autosomal recessive genetic disorder characterized by a deficiency of the lysosomal enzyme galactosylceramidase, GALC (Austin et al. 1970; Suzuki and Suzuki 1970). KD can manifest with different clinical presentations depending on the age of onset, ranging from early infantile, late‐infantile, to juvenile and adult‐onset forms (Bradbury et al. 2021; Mikulka and Sands 2016). As a progressive and neurodegenerative disorder, KD often leads to severe disability and a shortened lifespan (Bradbury et al. 2021; Mikulka and Sands 2016). Despite progress in understanding KD pathophysiology, it remains challenging to manage, and no curative therapy currently exists (Bradbury et al. 2021; Mikulka and Sands 2016).

GALC is essential for degrading galactosylceramide and psychosine, lipids enriched in myelin. Psychosine accumulation has been documented in KD patients (Im et al. 2001; Kobayashi et al. 1980; Svennerholm et al. 1980) and in animal models where it correlates with disease severity (Escolar et al. 2017; Guenzel et al. 2020; Ichioka et al. 1987; Igisu and Suzuki 1984; Shinoda et al. 1987; Tanaka et al. 1988), Although psychosine toxicity is central to KD pathology, work in mutant mouse models suggests that additional GALC‐dependent mechanisms also contribute to demyelination (Biswas et al. 2002; Nicaise et al. 2016; Weinstock et al. 2020) and neurodegeneration (Ezoe et al. 2000; Potter et al. 2013).

Lysosomes play a central role in autophagy, a homeostatic mechanism essential for degrading damaged organelles and proteins (Settembre and Ballabio 2014). Lysosomal dysfunction can impair autophagic flux and contribute to neurodegeneration (Udayar et al. 2022; Winslow and Rubinsztein 2008). Emerging research has unveiled a pivotal role of the autophagy‐lysosome pathway in modulating the pathophysiology of lysosomal storage disorders (LSDs) (Raben et al. 2007). The interaction between autophagy and LSDs is complex and bidirectional. On one hand, impaired lysosomal function resulting from LSD‐associated mutations can perturb autophagic flux (Lieberman et al. 2012), leading to compromised clearance of autophagosomes and exacerbating cellular stress. Conversely, the upregulation of autophagy represents a cellular response aimed at reducing the burden of accumulated substrates in LSD‐affected cells (Bartolomeo et al. 2017; Tornero‐Ecija et al. 2023). Supporting this concept, earlier cellular studies in KD models reported alterations in autophagy‐related pathways associated with GALC deficiency (Del Grosso et al. 2019; Ribbens et al. 2014), and more recent work using patient‐derived fibroblast models has further explored links between lysosomal dysfunction, cellular stress responses, and autophagy‐related signaling (Mezzena et al. 2023). Recent studies in the *twitcher* mice have tested whether enhancing autophagy improves disease outcome by starvation (Papini et al. 2023) as well as using broader autophagy inducers such as rapamycin and lithium, but success has been minimal (Del Grosso et al. 2024, 2022; Lin et al. 2023). However, how autophagy contributes to KD pathogenesis, particularly in Schwann cells, remains unclear.

Given that GALC‐deficient Schwann cells phenocopy key features of KD (Weinstock et al. 2020), we used this model to define autophagic changes during disease initiation. We observed early changes in autophagy‐associated proteins and an increase of autophagosome accumulation prior to overt myelin abnormalities, suggesting that GALC deficiency is associated with early alterations in autophagy‐related pathways in Schwann cells. To test whether autophagy contributes to disease progression, we deleted *Atg7*. ATG7 is an essential component of the canonical autophagy machinery and functions as an E1‐like activating enzyme required for autophagosome formation. Specifically, ATG7 participates in the ubiquitin‐like conjugation systems that regulate ATG12‐ATG5 complex formation and LC3 lipidation, a critical step required for autophagosome membrane expansion (Mizushima and Komatsu 2011). Because of its central role in canonical autophagy, conditional deletion of *Atg7* is widely used as a genetic strategy to disrupt ATG7‐dependent canonical autophagy in a cell type‐specific manner (Collier et al. 2021). Therefore, Schwann cell‐specific deletion of *Atg7* provides a genetic approach to determine whether canonical autophagy contributes to disease progression in GALC‐deficient Schwann cells. Our study shows that *Atg7* loss in *Galc* SC cKO mice worsened KD severity, with thinner myelin, an increase of unprocessed cytosolic LC3, and pronounced deficits in motor performance and nerve conduction. Our findings suggest that ATG7‐dependent autophagy pathways play a protective role during KD progression.

## 2 Materials and Methods

### 2.1 Animal Models

All mouse lines were backcrossed for at least 10 generations into the C57BL/6N background to ensure strain consistency (Charles River Laboratories International Inc.). Animals were housed in ventilated cages at 70°F ± 2°F, 30%–70% humidity, under a 12‐h light/dark cycle, with ad libitum access to food and water. All procedures were approved by the Institutional Animal Care and Use Committees of the University at Buffalo and Roswell Park Cancer Institute. *Galc* floxed mice were from (Weinstock et al. 2020). Schwann cell‐specific Cre recombination was achieved using *Mpz*‐Cre (P0‐Cre; JAX #017927) mice (Feltri et al. 1999). To generate Schwann cell‐specific *Atg7* conditional knockout mice (*Atg7* SC cKO), frozen sperm from *Atg7* floxed mice (RBRC02759), deposited by Dr. Masaaki Komatsu, was obtained from the RIKEN BioResource Center (Komatsu et al. 2005). For autophagy reporter experiments, LC3‐RFP‐EGFP transgenic mice (JAX #027139) were used (Li et al. 2014; Lin et al. 2014). Schwann cell‐specific GALC‐deficient mice (*Galc* SC cKO) were generated by crossing *Galc* floxed mice with *Mpz*‐Cre mice. *Atg7* SC cKO mice were crossed into the *Galc* SC cKO line to generate double conditional knockout mice, *Atg7‐Galc* SC cKO. Genotyping for all mouse lines was performed from genomic DNA obtained from ear punches or toe clippings following established protocols described in the original publications for each floxed allele and Cre line (Feltri et al. 1999; Komatsu et al. 2005; Li et al. 2014; Weinstock et al. 2020).

### 2.2 Immunohistochemistry

Sciatic nerves were fixed for 30 min in 4% PFA, washed 2× in PBS for 5 min, left overnight in 20% sucrose at 4°C and then frozen in optimal cutting temperature (OCT) compound. Longitudinal sections (10 μm) were cut on a cryostat (Leica CM 1950), collected on a slide, and circled with raw pap‐pen boundary. Sections were rehydrated in PBS for 10 min at room temperature (RT), blocked for 1 h at RT in a blocking buffer (3% BSA in PBS; 2% normal goat serum, 0.5% Triton X‐100). Primary antibodies were diluted in the blocking buffer and applied to sections for 1 h at RT in a humidified chamber. After washing 3× with PBS, secondary antibodies were similarly diluted in the blocking buffer and applied to sections for 1 h at RT. DAPI was added for 8 min at RT. Sections were then washed 3× with PBS and mounted in Vectashield mounting media (Vector Laboratories). Unless otherwise specified, all imaging was processed using Leica confocal microscopes (Leica TCS SP5 II & DMI 5000 CS).

### 2.3 Teased Fiber Preparation

Slides were prepared using Vectabond (Tespa). Nerves were fixed in 4% PFA for 30 min and then washed 3× with PBS. Nerves were de‐sheathed with forceps and a 27‐gauge needle. Bundles of fibers were separated in chilled PBS using 27‐gauge needles and then gently teased apart to single fibers on Tespa‐coated slides. Slides were allowed to dry for at least 1 h before staining. P7 and P14 teased sciatic nerve fibers from WT and *Galc* SC cKO, and P35 teased sciatic nerve fibers from WT, *Atg7* SC cKO, *Galc* SC cKO, and *Atg7‐Galc* SC cKO mice carrying the CAG‐RFP‐EGFP‐LC3 reporter were analyzed by confocal microscopy. RFP+/EGFP+ (yellow) puncta were defined as spatially overlapping RFP and EGFP signals that indicate autophagosome or unprocessed cytosolic LC3, whereas RFP+ only (red) puncta were defined as RFP‐positive structures lacking detectable EGFP signal. Total red puncta were calculated as the sum of yellow and red puncta. The fraction of yellow puncta relative to total red puncta was calculated for each animal. Multiple fields were analyzed from each animal, and puncta counts were normalized to the number of fields analyzed for each animal and are reported as puncta per field. Individual fields and puncta were treated as subsamples, and the animal was treated as the biological replicate for statistical comparisons.

### 2.4 Western Blot Analyses

Tissue or cells were lysed in RIPA buffer supplemented with phosphatase and protease inhibitors. For sciatic nerve: the samples were flash frozen in liquid nitrogen and pulverized with a pestle. The crushed powder was resuspended in lysis buffer. Lysates were centrifuged at 13,000 rpm for 10 min at 4°C and the supernatant was removed. Protein concentration was determined with the BCA protein assay kit (Thermo‐Fisher Scientific) according to the manufacturer's instructions. Samples were prepared with 4× Laemmli buffer and lysis buffer. 5–20 μg of protein were resolved using SDS‐polyacrylamide gel electrophoresis (SDS‐PAGE) under denaturing conditions with a mini‐Protean II gel electrophoresis apparatus and included a Precision Plus Standard Protein Dual color from (Bio‐Rad) to enable band size identification. Separated proteins were then transferred to an activated PVDF blotting membrane, in a mini gel transfer tank. Non‐specific binding sites on the membrane were blocked for 1 h at RT using 5% non‐fat dry milk in TBS/0.05% Tween 20. Primary antibodies in 3% BSA in TBS/0.05% Tween 20 (TBST) were incubated overnight at 4°C. Membranes were washed in TBST 3× for 5 min and incubated for 1 h at RT with secondary antibody. Secondary antibodies were either horseradish peroxidase‐conjugated (HRP) secondary antibody diluted in 5% milk TBST or Licor secondary fluorescent antibody diluted in Licor Odyssey buffer. Blots incubated with HRP‐conjugated secondary antibody were developed with ECL chemiluminescent reagent with film on a Chemi‐doc apparatus (Bio‐Rad), while Licor antibodies were developed using the Odyssey CLx apparatus (Li‐Cor). Full, uncropped western blot images are provided in Figure S3.

### 2.5 Antibodies

For western blot analyses the following antibodies were used: *Primary:* GAPDH, Sigma‐Aldrich #G9545; LC3B, Cell Signaling Technology #2775S; MBP, BioLegend #836504, Myelin Protein Zero, Aves #PZO; ATG7, Cell Signaling Technology #8558; ATG5, Cell Signaling Technology #12994; p62, Enzo #BML‐PW9860‐0100; LAMP1, Abcam #ab24170; and MAG, Zymed #34–6200. *Secondary*: Donkey anti‐Chicken HRP, Abcam #ab16349; Donkey anti‐Rabbit HRP, Novus #NB7185; 800CW Donkey anti‐Chicken, Licor #926‐32218; 800CW Goat anti‐Rabbit, Licor #926‐32211; 670CW Goat anti‐Rabbit, Licor #926‐68071; 670CW Goat anti‐Mouse, Licor #926‐68070. For immunohistochemical analysis the following antibodies were used. *Primary:* F4/80, Bio‐Rad #MCA497GA. *Secondary:* Jackson ImmunoResearch—Donkey Anti‐Rat IgG Cy3.

### 2.6 Nerve Conduction Velocity Analysis

Mice (35 days or 2 months) were anesthetized with 2,2,2 tribromoethanol (Sigma) 10 mg/mL in H_2_O and placed under a heating lamp. The sciatic nerve motor conduction velocity was obtained with steel monopolar needle electrodes: a pair of stimulating electrodes was inserted subcutaneously near the nerve at the ankle; and a second pair of electrodes was placed at the sciatic notch, and a third pair over the dorsum of the spine, in order to obtain distal, proximal, and spinal sites of stimulation along the nerve. The compound motor action potential (CMAP) was recorded with an active electrode inserted in muscles in the middle of the paw and a reference needle in the skin between the first and second digits.

### 2.7 Rotarod Analysis

Rotarod analyses were performed as previously described (Della‐Flora Nunes et al. 2017). All mice were tested in two sessions of three trials each per day (6 h rest between the two daily sessions) for two consecutive days. Analyses measuring 35‐day old animals occurred at P34 and P35; those measuring P60 animals occurred at P59 and P60. Only naïve mice were used for Rotarod tests (no training occurred prior to Trial 1). Rotarod conditions were set to an acceleration of 5 rotations per minute, starting at a minimum velocity of 4 rotations per minute and accelerating to a maximum velocity of 40 rotations per minute. Each trial consisted of one acclimating run that was not scored. The next three runs were recorded and averaged. Each run was stopped when the mouse fell or passed completely underneath the rod (180 degrees of rotation).

### 2.8 Semithin Morphology and Electron Microscopy

Nerves were dissected and fixed by immersion in 2% glutaraldehyde at least overnight. Nerves were washed in phosphate buffer 0.12 M pH 7.4 at RT and incubated in osmium tetroxide 1% in phosphate buffer 0.12 M pH 7.4 for 2 h at RT in the dark. Nerves were then washed twice in phosphate buffer 0.12 M pH 7.4, dehydrated in increasing EtOH concentrations (50%, 70%, 90%, and 100%) followed by incubation in propylene oxide. Propylene oxide was then evaporated, and nerves were embedded in Epon 100%. Resin was polymerized at 60°C overnight. Nerves were then cut into 1 μm semithin sections or 80–85 nm ultrathin sections. Semithin sections were stained with 2% toluidine blue in 0.12 M phosphate buffer, pH 7.4. Ultrathin sections were stained with saturated uranyl acetate (in dH2O) and lead citrate for 5 min each. Images for semithin were acquired with a ×100 objective on a Leica DM 6000 microscope running the Q‐Capture Pro V7.0.4324.5 software (QImaging Inc.) while for EM a FEI Tecnai G2 Spirit bio twin electron microscope was used. For quantification of myelin abnormalities, all axons in cross section of reconstructed sciatic nerves were counted. Axon density was quantified from reconstructed sciatic nerve cross‐sections by dividing the total number of axons by the analyzed nerve cross‐sectional area and is reported as axons/mm^2^. For the original P60 G‐ratio analysis, randomly selected EM fields acquired at 2900× were analyzed from WT, *Galc* SC cKO, and *Atg7‐Galc* SC cKO sciatic nerves. G‐ratio was calculated as axon diameter divided by the total myelinated fiber diameter. A total of 100–150 myelinated axons were measured per animal, with three animals analyzed per genotype. Individual axons were treated as subsamples nested within each animal, and the animal was treated as the biological replicate for statistical comparisons. Individual axon measurements were retained in G‐ratio‐versus‐axon‐diameter scatterplots for descriptive visualization. Because axons ≥ 7 μm were sparsely represented in the sampled EM fields, whole‐nerve semithin cross‐sections from P60 WT, *Galc* SC cKO, and *Atg7‐Galc* SC cKO nerves were additionally analyzed to improve sampling of large‐caliber axons. Semithin images acquired at 100× were stitched without rescaling and calibrated at 0.08625 μm/pixel. Axon and myelinated fiber boundaries were identified from the toluidine blue‐stained cross‐sections, and equivalent‐circle diameters were calculated from the corresponding segmented areas. Semithin G‐ratio was calculated as axon equivalent diameter divided by myelinated fiber equivalent diameter. Measurements were excluded when the axon‐myelin boundary or outer myelin contour could not be reliably resolved, when the outer contour merged with an adjacent fiber, or when the candidate represented an ambiguous or nonaxonal structure. All candidate axons ≥ 7 μm included in the analysis were visually reviewed to confirm the presence of a genuine, myelinated axon and a resolvable myelin boundary. For axon‐caliber‐dependent analysis, axons were grouped into 1‐μm diameter bins. EM‐derived measurements were used for diameter bins below 7 μm, whereas semithin‐derived measurements were used for the 7–8 and > 8 μm bins. Within each diameter bin, individual axon G‐ratios were first averaged within each animal, and these animal‐level means were used for genotype comparisons. Three animals per genotype were analyzed, except where an individual animal contained no axons within a particular diameter bin. Unless stated otherwise, quantifications were performed using Fiji v2.14.0/1.54f (NIH); the whole‐nerve semithin G‐ratio analysis was performed using the image‐analysis workflow described above. Onion bulb‐like structures were identified in P60 semithin sciatic nerve cross‐sections by the presence of concentric Schwann cell processes surrounding an axon. Quantification was performed across sampled nerve fields and summarized at the animal level, with individual fields and structures treated as subsamples within each animal. Three animals per genotype were analyzed. For analysis of axonal morphology, axons in P60 sciatic nerve electron micrographs were classified as morphologically abnormal when they exhibited abnormal axoplasmic morphology with accumulation of intracellular organelles relative to the normal axoplasmic architecture. Morphologically abnormal axons were quantified for each animal, with individual axons treated as subsamples and the animal treated as the biological replicate. For quantification of membrane‐bound myelin‐containing structures, structures were identified within the Schwann cell cytoplasm based on the presence of discrete membrane‐bound inclusions containing recognizable multilamellar or compact myelin‐like membrane material that was spatially distinct from the normal compact myelin sheath surrounding the axon. Each spatially discrete inclusion was counted as an individual structure. Quantification was performed at the animal level, with individual Schwann cells and structures treated as subsamples within each animal.

### 2.9 Total RNA Preparation and Real Time Quantitative PCR (RT‐qPCR)

Total RNA was extracted from homogenized sciatic nerve tissue using TRIzol reagent (Invitrogen). Subsequently, cDNA was synthesized using the SuperScript IV Reverse Transcriptase kit (Invitrogen) following the manufacturers protocol. Quantitative PCR (RT‐qPCR) was performed using SYBR Green reagents (Bio‐Rad), with target gene expression levels normalized to beta‐actin as an internal control. Relative fold changes in gene expression were determined using the ∆∆*C*_*t*_ method (Livak and Schmittgen 2001). Oligonucleotide primer sequences used for the qPCR reactions were TNFa‐sense; 5′‐CCACCACGCTCTTCTGTCTA‐3′, TNFa‐antisense; 5′‐CACTTGGTGGTTTGCTACGA‐3′ (amplicon size = 173 bp), MCP‐1‐sense; 5′‐CTTCTGGGCCTGCTGTTCA‐3′, MCP‐1‐antisense; 5′‐CAGCCTACTCATTGGGATCA‐3′ (amplicon size = 126 bp), MMP3‐sense; 5′‐GGAAATCAGTTCTGGGCTATACGA‐3′, MMP3‐antisense; 5′‐TAGAAATGGCAGCATCGATCTTC‐3′ (amplicon size = 112 bp), GADD34‐sense; 5′‐GGTGGTCCAGCTGAGAATGA‐3′, GADD34‐antisense; 5′‐TCTTCCGTGGCTTGATGTTC‐3′ (amplicon size = 137 bp), and beta‐actin‐sense; 5′‐GACAGGATGCAGAAGGAGATTAC‐3′, beta‐actin‐antisense; 5′‐CTGCTTGCTGATCCACATCT‐3′ (amplicon size = 150 bp).

### 2.10 Statistical Analysis

Data collection and analyses were performed blind to genotypes and conditions of experiments. For morphometric analyses containing multiple axons or imaging fields per animal, the animal was considered the biological replicate, while individual axons, fields, or structures were treated as subsamples. Statistical tests of experiments are included in figure legends. GraphPad Prism 10 software was used for Student's *t*‐test, one‐way ANOVA, and two‐way ANOVA as reported in the legend of every figure. Statistical significance was defined as *p* < 0.05, with significant differences denoted by asterisks. *p < 0.05, **p < 0.01; ***p < 0.001; ****p < 0.0001. Data throughout text and figures are presented as mean ± SEM unless otherwise specified.

## 3 Results

### 3.1 Autophagy‐Associated Protein Levels Are Changed During Disease Initiation in the KD PNS

To study autophagic changes during KD pathology initiation, we employed our previously validated *Galc* SC cKO mouse model where *Galc* is conditionally ablated from SCs resulting in a PNS KD pathology (Weinstock et al. 2020). In that study, we investigated the pathological phenotype of *Galc* SC cKO nerves at postnatal day 35 (P35) using semithin microscopy and immunohistochemistry (IHC). Compared to normal wild‐type (WT) nerves, *Galc* SC cKO sciatic nerves exhibited edema and autophagic accumulation of myelin at P35 (Weinstock et al. 2020). Following that study, to determine the initiation of pathology, we collected sciatic nerves from WT and *Galc* SC cKO mice at various ages (P7, P14, P21, and P28) to perform semithin microscopy. Analysis of myelin abnormalities, including myelin infoldings, outfoldings, decompaction, and onion bulbs, revealed that sciatic nerves of *Galc* SC cKO mice develop normally during the first two postnatal weeks (P7 and P14), where both WT and *Galc* SC cKO axons undergo radial sorting and myelination without significant differences. However, morphological differences became evident after P14, with *Galc* SC cKO mice exhibiting nerve edema and minor myelin abnormalities (Figure 1A,B). Quantification of nerve area showed significant differences at P21 and P28, suggesting that nerve edema starts after P14 in the PNS of KD model (Figure 1A,B). Myelin abnormalities, such as myelin infoldings, outfoldings, decompaction, and onion bulbs were significantly increased in *Galc* SC cKO nerves at P28 (Figure 1C). These findings collectively indicate that pathology initiates after P14 (between P14 and P21) in *Galc* SC cKO mice, followed by a progressive worsening of the nerve phenotype.

**FIGURE 1:**
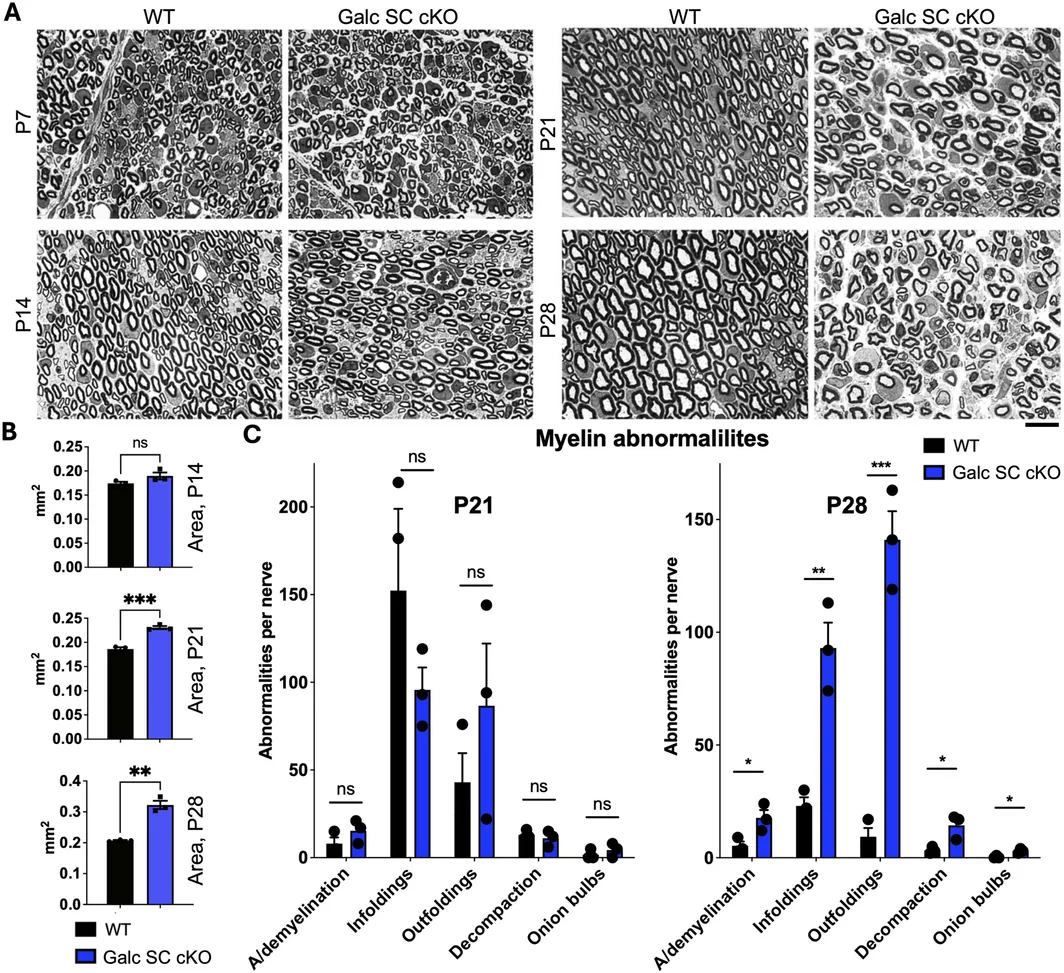
Characterization of Krabbe disease initiation in sciatic nerves of *Galc* SC cKO mice. (A) Representative semithin cross section images of sciatic nerves of WT and *Galc* SC cKO at P7, P14, P21, and P28. Scale bar = 10 μm. (B) Quantification of sciatic nerve cross sectional area analyses at P14, P21, and P28. Three animals per genotype were analyzed. WT and *Galc* SC cKO groups were compared at each age using Student's *t*‐test. (C) Quantification of a/demyelinated axon fibers, myelin infoldings, myelin outfoldings, myelin decompactions, and onion bulbs, per whole sciatic nerve cross section at P21 and P28. Three animals per genotype were analyzed. WT and *Galc* SC cKO groups were compared at each age using Student's *t*‐test. *p < 0.05; **p < 0.01; ***p < 0.001; ****p < 0.0001; n.s. = not significant. Data are presented as mean ± SEM.

SCs use autophagy machinery to degrade/clear myelin debris (Gomez‐Sanchez et al. 2015; Jang et al. 2015), and autophagy was found to be upregulated in sciatic nerves during mouse development (Jang et al. 2015) when SCs are actively myelinating the axon fibers, suggesting that autophagy might play an important role during PNS myelin development. Thus, insults in the autophagy pathway during this time might affect normal development and myelination of the sciatic nerve. After confirming the disease initiation time point at around P14, we next examined the protein expression levels of autophagy markers, ATG5, ATG7, WIPI2, and LC3‐II/LC3‐I, in the sciatic nerves of WT and *Galc* SC cKO mice at P14 and P21 using western blot (WB) analysis (Figure 2A). ATG7 regulates autophagosome formation by recruiting and conjugating other ATG proteins such as ATG5 and ATG12, as well as by driving LC3 lipidation from LC3‐I to LC3‐II form, with LC3‐II being associated as a marker for autophagosomes (Collier et al. 2021). Interestingly, the ATG7 and LC3‐II/LC3‐I ratio was significantly increased in the *Galc* SC cKO nerves at P21, but not at P14 (Figure 2A). Separate quantification of LC3‐I and LC3‐II normalized to GAPDH showed a significant reduction in LC3‐I at P21 in *Galc* SC cKO nerves, whereas LC3‐II showed a higher mean that did not reach statistical significance (Figure 2A). WD‐repeat PtdIns(3)P effector protein 2 (WIPI2) is also used as an autophagy marker as it binds to ATG16L1, recruiting the ATG12‐5‐16 L1 complex for LC3 lipidation and autophagosome formation (Dooley et al. 2014). Our data show that WIPI2 is significantly increased at P14 in the *Galc* SC cKO nerves, and the increase remained apparent at P21, although the difference was not significant (Figure 2A). p62, also known as sequestosome 1 (SQSTM1), is an autophagy‐associated cargo receptor that binds ubiquitinated proteins and can be degraded through the autophagy‐lysosome pathway. However, its steady‐state abundance may also be influenced by transcription, protein synthesis, tissue composition, and other degradative pathways. Intriguingly, p62 levels were substantially increased in the sciatic nerves of *Galc* SC cKO at both P14 and P21, indicating altered p62 homeostasis in *Galc* SC cKO sciatic nerves (Figure 2A). ATG5 levels were unchanged at both P14 and P21 (Figure 2A). Collectively, these findings demonstrate altered steady‐state levels of autophagy‐associated proteins at P14 and P21, though it is not clear if autophagic flux is also affected in KD since it has not been tested with direct lysosomal inhibition.

**FIGURE 2:**
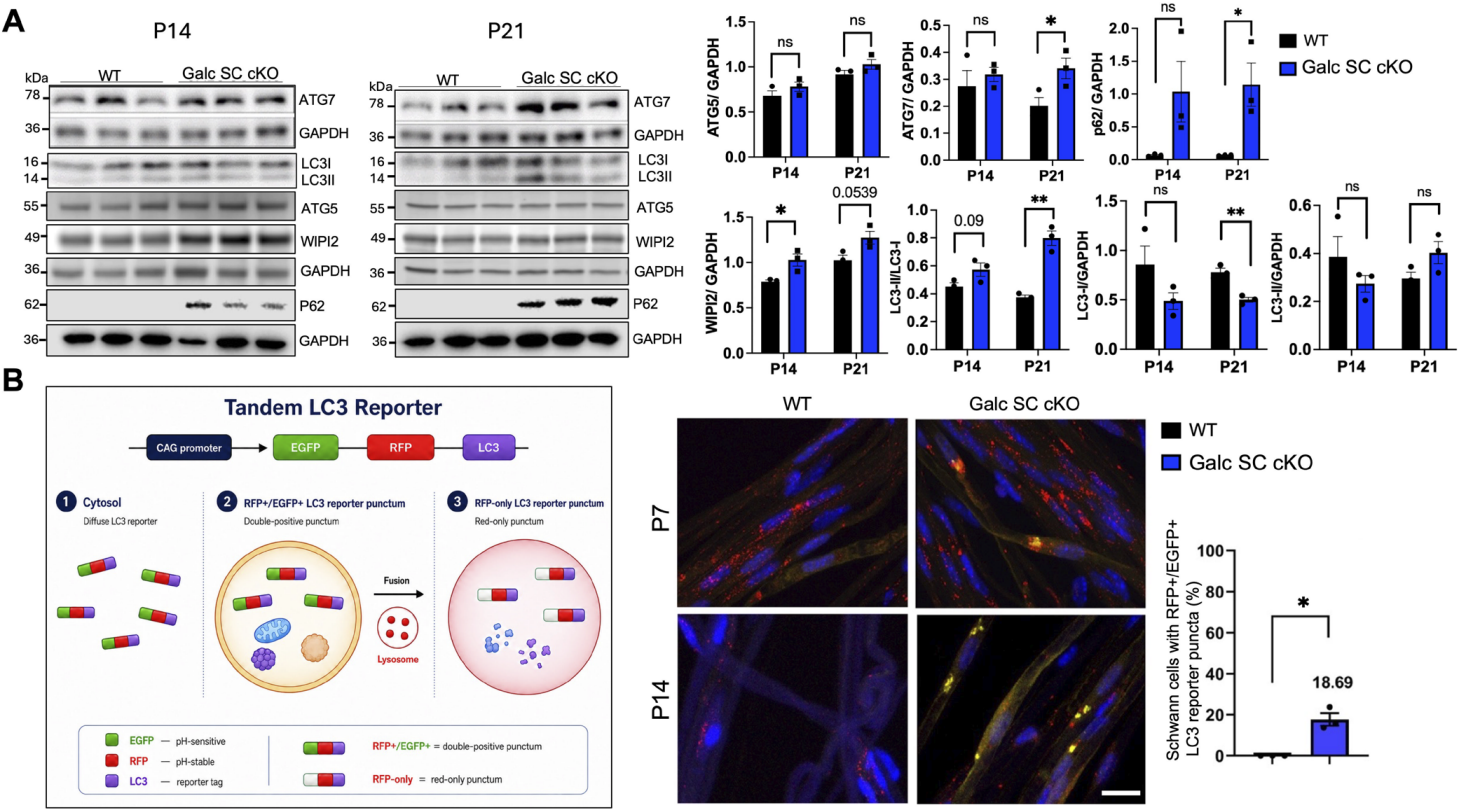
Autophagy‐associated protein levels in WT and *Galc* SC cKO nerves during development and myelination. (A) Western blot analysis of ATG5, ATG7, WIPI2, p62, LC3‐II/LC3‐I ratio, LC3‐I/GAPDH, and LC3‐II/GAPDH in WT and Galc SC cKO sciatic nerves at P14 and P21. GAPDH was used as the loading control. Molecular weights are indicated in kDa. Three animals per genotype were analyzed. WT and *Galc* SC cKO groups were compared at each age using Student's *t*‐test. (B) Schematic of tandem RFP‐EGFP‐LC3 reporter and representative images of teased sciatic nerve fibers from WT and *Galc* SC cKO mice at P7 and P14. Schwann cells were identified by their characteristic elongated morphology along individual myelinated axons. EGFP (green) and RFP (red) signals correspond to the tandem LC3 reporter. RFP+/EGFP+ puncta were classified as double‐positive LC3 reporter puncta (yellow), whereas RFP+ only signals were classified as red LC3 puncta. Quantification shows the percentage of Schwann cells containing yellow LC3 puncta. Scale bar = 10 μm. Three animals per genotype were analyzed. WT and *Galc* SC cKO groups were compared at each age using Student's *t*‐test. *p < 0.05; **p < 0.01; ***p < 0.001; ****p < 0.0001; n.s. = not significant. Data are presented as mean ± SEM.

To assess the autophagosome level in our KD model, we crossed the *Galc* SC cKO model with a pH‐sensitive transgenic CAG‐RFP‐EGFP‐LC3 reporter mouse model (Li et al. 2014; Lin et al. 2014). In this tandem reporter system, RFP+/EGFP+ (yellow) puncta represent double‐positive, relatively non‐acidified LC3 reporter structures, whereas RFP + only (red) puncta represent LC3 reporter structures in which the acid‐sensitive EGFP signal has been quenched (Figure 2B). Confocal microscopy analysis of single fibers from dissected sciatic nerves revealed an increased percentage of SCs containing yellow LC3 reporter puncta in *Galc* SC cKO mice as early as P7, with the increase progressing with age (Figure 2B). LC3 reporter puncta were analyzed in teased sciatic nerve fiber preparations, which preserve individual myelinated Schwann cell‐axon units. In this preparation, LC3 puncta localize along the elongated Schwann cell cytoplasm surrounding axons, allowing identification of Schwann cells based on morphology. Macrophages are typically washed out from teased nerve fibers during the preparation of specimens for this analysis. The increased prevalence of yellow LC3 puncta is consistent with increased autophagosome abundance in KD Schwann cells; however, whether this reflects increased formation, impaired clearance, or defective autophagosome‐lysosome fusion cannot be distinguished from these data alone.

### 3.2 Atg7 Deletion in SCs Does Not Affect Normal Development and Myelination of the Sciatic Nerve

To evaluate the role of ATG7 in Schwann cell function and myelin regulation in the PNS, we generated *Atg7* SC cKO mice by crossing *Atg7* floxed mice with *Mpz*‐Cre transgenic mice. We confirmed efficient *Atg7* deletion using WB of primary Schwann cells isolated from WT and *Atg7* SC cKO mice. To assess the functional consequence of ATG7 loss on autophagy, we examined ATG7 protein levels and LC3‐I to LC3‐II lipidation. ATG7 protein levels were markedly reduced in *Atg7* SC cKO Schwann cells compared with WT controls. Consistent with impaired canonical LC3 lipidation following *Atg7* deletion, the LC3‐II/LC3‐I ratio was significantly decreased, and LC3‐II normalized to GAPDH was also significantly reduced, whereas LC3‐I/GAPDH was not significantly different between genotypes (Figure 3A). Together, these findings confirm efficient ATG7 depletion and are consistent with reduced canonical LC3 lipidation in *Atg7*‐deficient Schwann cells. To assess developmental myelination, myelin morphology, and myelin abnormalities during the active myelination period, we collected sciatic nerves from WT and *Atg7* SC cKO mice and performed semithin microscopy at P14 (Figure 3B,C). Deleting *Atg7* in SCs did not affect myelination of sciatic nerve at P14. Similarly, we did not observe a change in myelin abnormalities between WT and *Atg7* SC cKO mice at this age. We further collected sciatic nerves from WT and *Atg7* SC cKO mice at P60 and conducted semithin microscopy to assess myelin morphology during the maintenance period (Figure 3B). Consistently, myelin phenotype in adult WT and *Atg7* SC cKO mice at P60 was unchanged. Collectively, our data suggest that *Atg7* deletion in SCs does not affect normal myelination and myelin maintenance of sciatic nerves.

**FIGURE 3:**
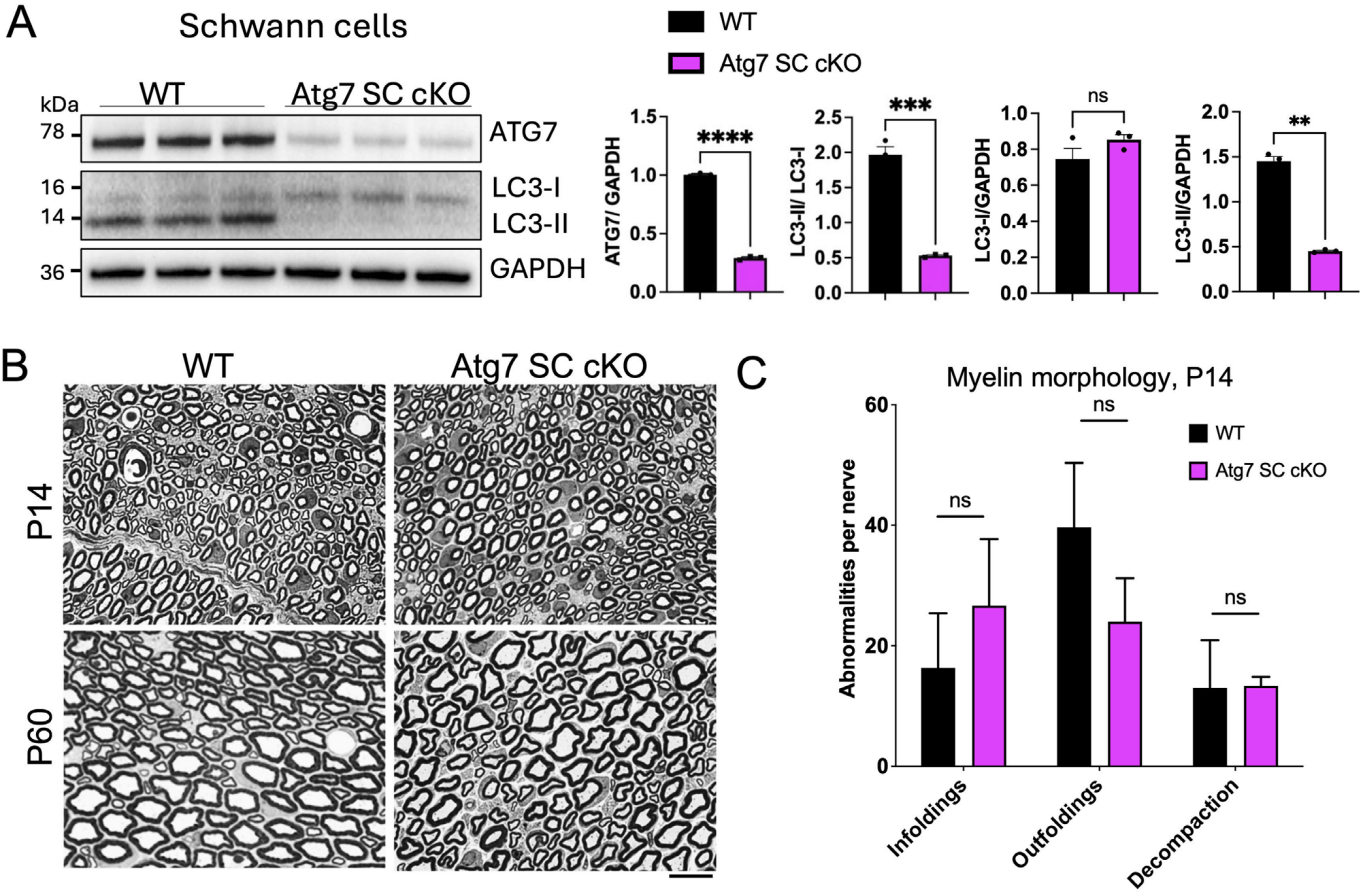
*Atg7* ablation in SCs does not affect development and myelination of sciatic nerve. (A) Representative Western blots and quantification of ATG7 and the LC3‐I and LC3‐II in primary SCs cultured from WT and Atg7 SC cKO mice. Quantification includes ATG7/GAPDH, LC3‐II/LC3‐I, LC3‐I/GAPDH, and LC3‐II/GAPDH. GAPDH was used as the loading control. Molecular weights are indicated in kDa. Three animals per genotype were analyzed. WT and *Atg7* SC cKO groups were compared using Student's *t*‐test. (B) Representative semithin cross sections of sciatic nerves from WT and Atg7 SC cKO at P14 and P60. Scale bar = 10 μm. C. Quantification of myelin infoldings, myelin outfoldings, and myelin decompactions per sciatic nerve cross section at P14. Three animals per genotype were analyzed. WT and *Atg7* SC cKO groups were compared using Student's *t*‐test. *p < 0.05; **p < 0.01; ***p < 0.001; ****p < 0.0001; n.s. = not significant. Data are presented as mean ± SEM.

### 3.3 Atg7 Deletion in KD SCs Reduces Myelin Sheath Thickness During Disease Progression

To evaluate the role of ATG7 in SCs in PNS KD model, we deleted *Atg7* from SCs by crossing *Atg7* floxed mice with *Galc* SC cKO mice. We hypothesized that impairing autophagy by deleting *Atg7* in *Galc* SC cKO mice would result in a worsening of the disease pathology. We collected sciatic nerves at P14, P35, and P60 and performed semithin microscopy to assess myelination and myelin morphology during development and maintenance periods (Figure 4A). We observed a similar myelin phenotype between *Galc* SC cKO and *Atg7‐Galc* SC cKO sciatic nerves at P14 and P35. We then performed a G‐ratio analysis at P60 using EM images to assess myelin thickness between WT, *Galc* SC cKO, and *Atg7‐Galc* SC cKO sciatic nerve fibers. Individual axon measurements were retained in the G‐ratio versus axon‐diameter scatterplot for visualization, whereas statistical comparisons were performed using animal‐level mean G‐ratios, with individual axons treated as subsamples within each animal. Mean G‐ratio was 0.669 ± 0.006 in WT, 0.752 ± 0.001 in *Galc* SC cKO, and 0.789 ± 0.008 in *Atg7‐Galc* SC cKO nerves (*n* = 3 per genotype). One‐way ANOVA followed by Tukey's multiple‐comparisons test showed a significantly increased G‐ratio in *Galc* SC cKO compared with WT and a further increase in *Atg7‐Galc* SC cKO compared with *Galc* SC cKO. Analysis across axon‐diameter bins demonstrated increased G‐ratios in *Galc* SC cKO and *Atg7‐Galc* SC cKO nerves compared with WT, with the greatest differences becoming apparent among larger‐caliber axons. Because axons ≥ 7 μm were sparsely represented in the EM dataset, whole‐nerve semithin sections were additionally analyzed to extend sampling of large‐caliber fibers. The corresponding animal‐level size‐bin analysis demonstrated that myelin thinning in *Galc* SC cKO and *Atg7‐Galc* SC cKO nerves was also evident among large‐caliber axons (Figure 4C). Representative high‐magnification EM images of similar‐caliber axons further illustrate the difference in myelin sheath thickness between *Galc* SC cKO and *Atg7‐Galc* SC cKO nerves (Figure 4B). Onion bulb‐like structures were also observed in P60 *Galc* SC cKO and *Atg7‐Galc* SC cKO sciatic nerves but were absent from WT nerves, presumably due to the impaired myelin maintenance. Animal‐level quantification demonstrated a significant increase in onion bulb‐like structures in both *Galc* SC cKO and *Atg7‐Galc* SC cKO nerves compared with WT. Whereas *Atg7* deletion marginally increased their prevalence beyond that observed in *Galc* SC cKO nerves, the change was not statistically significant (Figure 4D). Additional western blot analyses of myelin proteins including MBP, MPZ, and MAG with protein lysates from P60 sciatic nerves showed that the effect of *Atg7* ablation in KD SCs was not discernible to affect overall myelin protein levels (Figure 5A,B; *Galc* SC cKO vs. *Atg7‐Galc* SC cKO). Collectively, our data suggest that deletion of *Atg7* in *Galc* SC cKO mice exacerbates myelin thinning by P60, including in large‐caliber axons.

**FIGURE 4:**
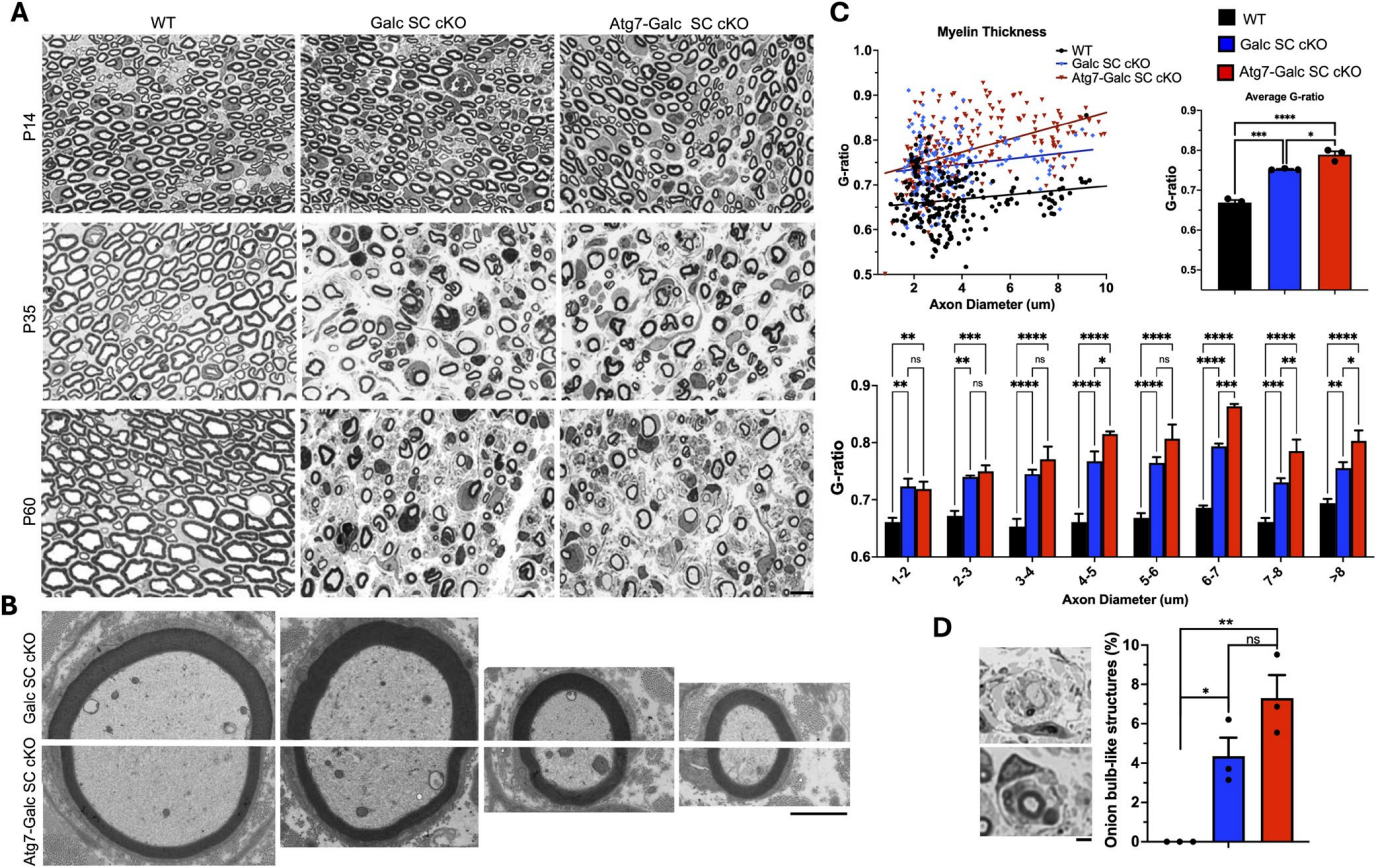
*Atg7* ablation in KD SCs exacerbates myelin thinning in P60 sciatic nerves. (A) Representative semithin cross sections of sciatic nerves from WT, *Galc* SC cKO, and *Atg7‐Galc* SC cKO at P14, P35, and P60. Scale bar = 10 μm. (B) Representative high‐magnification electron micrographs shown as split composite images comparing Galc SC cKO and Atg7‐Galc SC cKO myelinated axons across a range of axon calibers. Each composite was assembled from separate axons of approximately similar caliber from the two genotypes to facilitate visual comparison of myelin thickness. Images are shown at the same scale and were used for qualitative comparison only. Scale bar = 2 μm. (C) G‐ratio analysis of P60 sciatic nerve axons from WT, *Galc* SC cKO, and *Atg7‐Galc* SC cKO mice. The G‐ratio versus axon‐diameter scatterplot displays individual axon measurements for visualization, with lines representing linear regression for each genotype. Individual axons were treated as subsamples and were not considered independent biological replicates for statistical comparisons. The G‐ratio bar‐graph shows the mean G‐ratio for each animal, with each dot representing one animal (*n* = 3 animals/genotype). Approximately 100–150 myelinated axons were quantified per animal. The axon‐diameter‐bin graph shows animal‐level mean G‐ratios within each diameter range; axon measurements were first averaged within each animal, with the animal treated as the biological replicate. EM‐derived measurements were used for axons < 7 μm, whereas whole‐nerve semithin analysis was used to extend sampling of large‐caliber axons ≥ 7 μm. Overall G‐ratio comparisons were analyzed by one‐way ANOVA followed by Tukey's multiple‐comparisons test. Diameter‐dependent G‐ratio comparisons were analyzed using two‐way ANOVA followed by Tukey's multiple‐comparisons test between genotypes within each diameter bin. (D) Representative P60 semithin sciatic nerve images and quantification of onion bulb‐like structures across nerve cross section in WT, *Galc* SC cKO, and *Atg7‐Galc* SC cKO mice. Onion bulb‐like structures were identified by concentric Schwann cell processes surrounding myelinated axons. Scale bar = 5 μm. Quantification was summarized at the animal level, with individual structures/fields treated as subsamples and each dot representing one animal (*n* = 3 animals/genotype). One‐way ANOVA followed by Tukey's multiple‐comparisons test were used for comparison. *p < 0.05; **p < 0.01; ***p < 0.001; ****p < 0.0001; n.s. = not significant. Data are presented as mean ± SEM.

**FIGURE 5:**
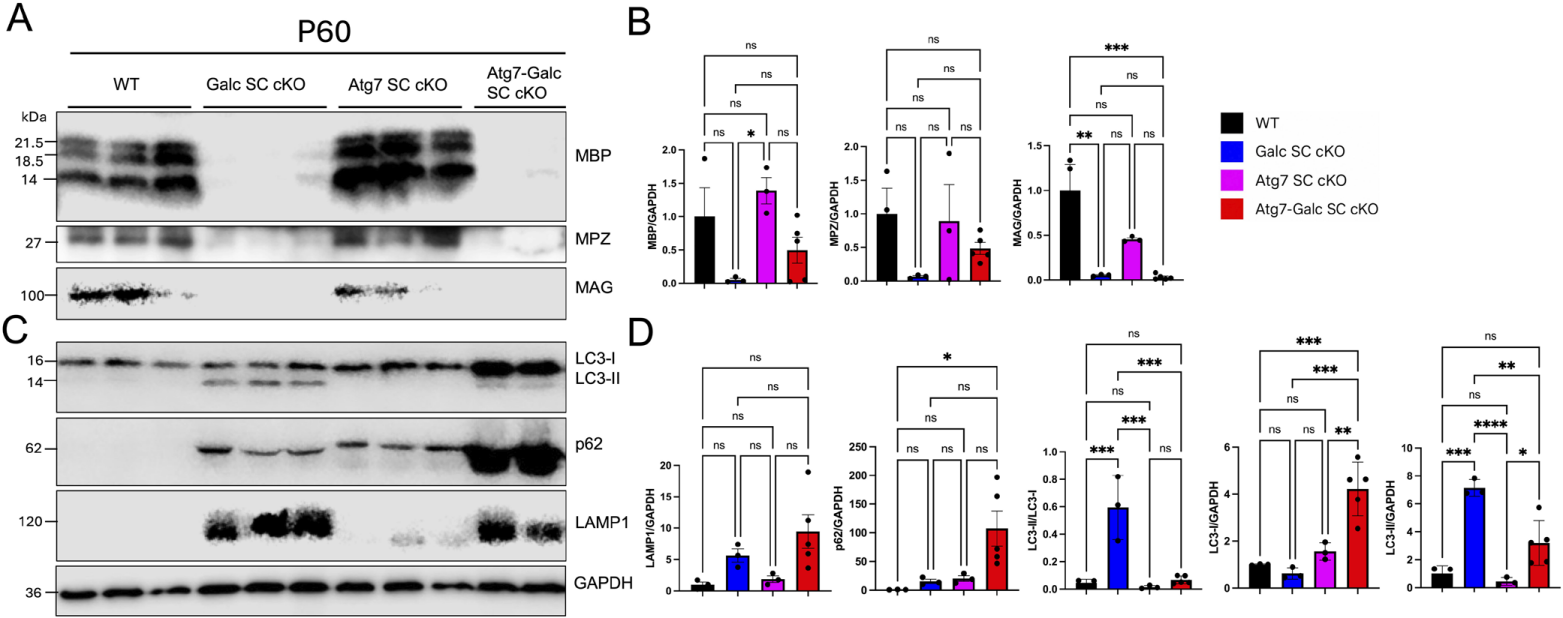
*Atg7* ablation in KD SCs alters steady‐state autophagy‐associated protein levels without affecting myelin protein levels. (A) Representative western blots for myelin proteins MBP, MPZ, and MAG in P60 sciatic nerves from WT, *Galc* SC cKO, *Atg7* SC cKO, and *Atg7‐Galc* SC cKO mice. (B) Quantification of MBP, MPZ, and MAG normalized to GAPDH. (C) Western blots for LC3‐I, LC3‐II, p62, and LAMP1 in P60 sciatic nerves. GAPDH was used as the loading control for (A) and (C). Molecular weights are indicated in kDa. (D) Quantification of p62/GAPDH, LAMP1/GAPDH, LC3‐II/LC3‐I, LC3‐I/GAPDH, and LC3‐II/GAPDH. Groups were compared by One‐way ANOVA. Each dot represents one animal. *p < 0.05; **p < 0.01; ***p < 0.001; ****p < 0.0001; n.s. = not significant. Data are presented as mean ± SEM.

### 3.4 Atg7 Deletion in KD SCs Increases Unprocessed LC3 in Schwann Cells but Does Not Affect Macrophage Abundance or Axonal Morphology in the Sciatic Nerve

To determine how *Atg7* deletion altered autophagy‐associated proteins in GALC‐deficient Schwann cells, we assessed steady‐state levels of p62, LC3‐I, LC3‐II, and LAMP1 at P60. Compared with *Galc* SC cKO nerves, *Atg7‐Galc* SC cKO nerves showed higher p62 and LAMP1 levels, though neither difference reached statistical significance (p62, *p* = 0.0632; LAMP1, *p* = 0.5604) (Figures 5C,D and S1). Expression levels of LC3‐I and LC3‐II, normalized to GAPDH each, revealed that LC3‐I was significantly increased while LC3‐II was significantly decreased in *Atg7‐Galc* SC cKO nerves compared with *Galc* SC cKO. Also, the LC3‐II/LC3‐I ratio was significantly reduced in *Atg7‐Galc* SC cKO nerves compared with *Galc* SC cKO. These steady‐state changes are consistent with reduced canonical LC3 lipidation following *Atg7* deletion.

We also crossed the WT, *Atg7* SC cKO, *Galc* SC cKO, and *Atg7‐Galc* SC cKO mice with the pH‐sensitive transgenic CAG‐RFP‐EGFP‐LC3 reporter and analyzed LC3 positive autophagy at P35 (Figure 6A). The analysis was done in teased sciatic nerve fibers, which preserve individual myelinated Schwann cell‐axon units. In this preparation, LC3 puncta localize along the elongated Schwann cell cytoplasm surrounding axons, allowing identification of Schwann cells based on morphology. Interestingly, the percentage of Schwann cells containing yellow LC3 reporter puncta was increased in *Atg7*‐*Galc* SC cKO nerves compared with *Galc* SC cKO, indicating a further dysregulated autophagic pathway (Figure 6A). In addition, the proportion of yellow puncta relative to total red puncta was increased in *Atg7‐Galc* SC cKO nerves, though total red or red only puncta per field were not significantly different among genotypes (Figure S2). Because *Atg7* deletion impairs canonical LC3 lipidation, the precise biological identity of the yellow puncta observed in ATG7‐deficient Schwann cells remains unresolved and may include non‐lipidated LC3 aggregates or other LC3 associated structures.

**FIGURE 6:**
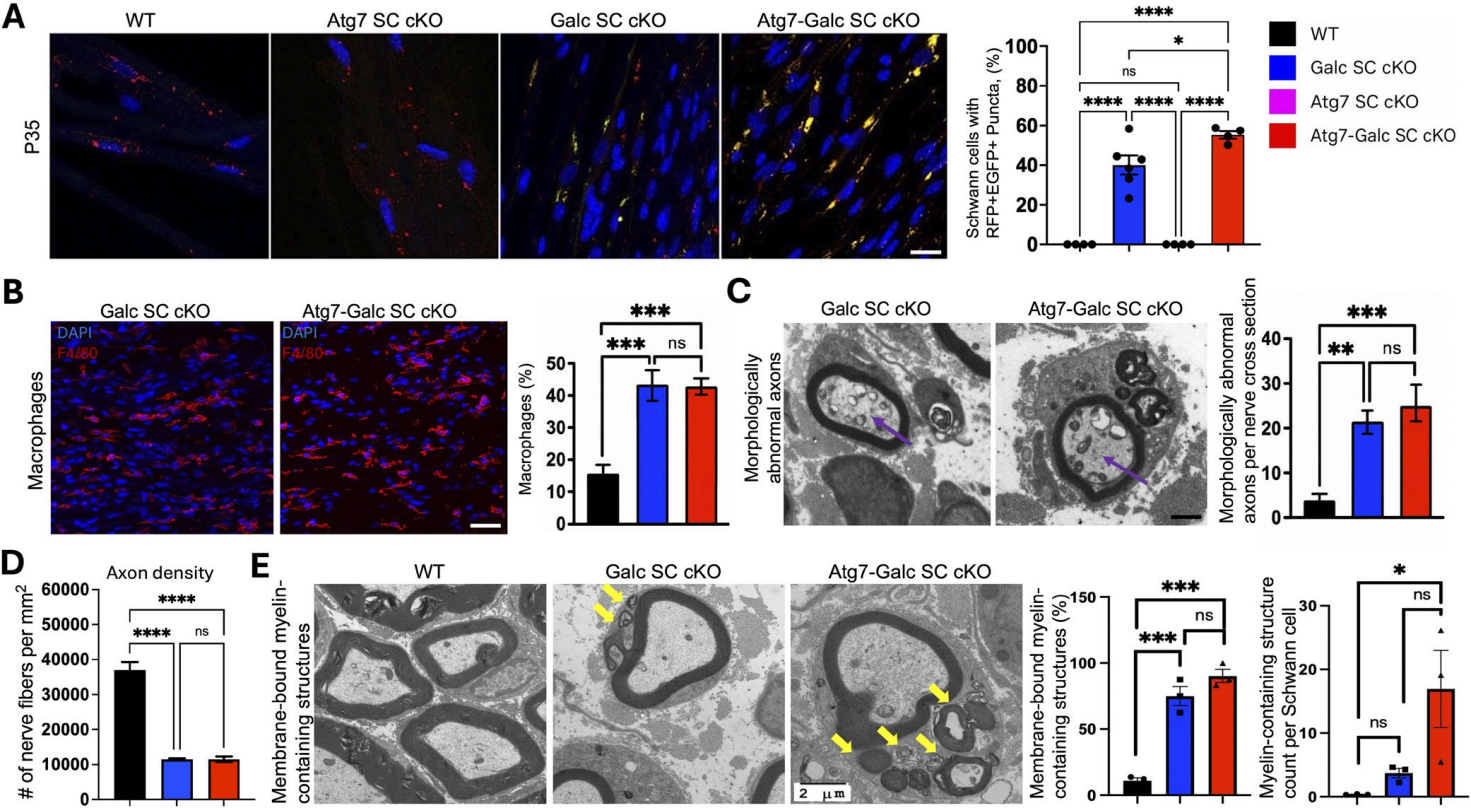
*Atg7* ablation in KD SCs alters LC3 reporter level without further increasing macrophage abundance or axonal pathology. (A) CAG‐RFP‐EGFP‐LC3 reporter analysis in P35 teased sciatic nerve fibers from WT, *Atg7* SC cKO, *Galc* SC cKO, and *Atg7*‐Galc SC cKO mice. Schwann cells were identified by their characteristic elongated morphology along individual myelinated axons. The percentage of Schwann cells containing yellow LC3 reporter puncta were quantified. Additional quantification of total red LC3 reporter puncta per field, yellow LC3 reporter puncta per field, red only LC3 reporter puncta per field, and the percentage of yellow puncta relative to total red puncta is shown in Figure S2. Puncta counts were normalized to the number of fields analyzed for each animal. WT, *Galc* SC cKO, and *Atg7‐Galc* SC cKO groups contained four animals each; the *Atg7* SC cKO group contained three animals. Scale bar = 10 μm. Each dot represents one animal. Groups were compared by ordinary one‐way ANOVA followed by Tukey's multiple‐comparisons test. (B) Representative confocal images and quantification of 10‐μm longitudinal sections of sciatic nerves from P60 WT, *Galc* SC cKO, and *Atg7‐Galc* SC cKO mice stained for F4/80 (macrophages) and DAPI. Scale bar = 50 μm. Three animals per genotype were analyzed. Groups were compared by one‐way ANOVA followed by Tukey's multiple‐comparisons test. (C) Representative EM images and quantification of morphologically abnormal axons in P60 sciatic nerve cross‐sections from WT, *Galc* SC cKO, and *Atg7‐Galc* SC cKO mice. Purple arrows indicate morphologically abnormal axons characterized by abnormal axoplasmic morphology with conspicuous intracellular organelle accumulation relative to normal axons. Scale bar = 1 μm. Three animals per genotype were analyzed. Groups were compared by one‐way ANOVA followed by Tukey's multiple‐comparisons test. (D) Axon density in P60 sciatic nerve cross‐sections from WT, *Galc* SC cKO, and *Atg7‐Galc* SC cKO mice. Axon density was calculated as the number of axons per mm^2^ of nerve cross‐sectional area. Three animals per genotype were analyzed, with each dot representing one animal. Groups were compared by one‐way ANOVA followed by Tukey's multiple‐comparisons test. (E) Representative EM images and quantification of membrane‐bound myelin‐containing structures within Schwann cell cytoplasm in P60 WT, *Galc* SC cKO, and *Atg7‐Galc* SC cKO sciatic nerves. Yellow arrows indicate membrane‐bound myelin‐containing structures, defined as discrete cytoplasmic inclusions containing recognizable multilamellar or compact myelin‐like membrane material distinct from the normal myelin sheath. Quantification shows the percentage of Schwann cells containing membrane‐bound myelin‐containing structures and the number of these structures per Schwann cell. Scale bar = 2 μm. Three animals per genotype were analyzed. Statistical comparisons were performed by one‐way ANOVA followed by Bonferroni's post hoc test. *p < 0.05; **p < 0.01; ***p < 0.001; ****p < 0.0001; n.s. = not significant. Data are presented as mean ± SEM.

As *Atg7‐Galc* SC cKO mice exhibited thinner myelin in their sciatic nerves at P60, we next asked whether this phenotype was accompanied by increased macrophage recruitment. We performed an IHC using the macrophage marker F4/80 in the longitudinal sciatic nerve sections from P60 *Galc* SC cKO and *Atg7‐Galc* SC cKO mice (Figure 6B). Macrophage abundance was similar between the two mutant genotypes, indicating that *Atg7* deletion did not further increase macrophage recruitment at this stage. However, these measurements assess macrophage abundance and do not exclude potential differences in macrophage activation, phagocytic activity, or inflammatory function. We next examined whether *Atg7* deletion further altered axonal pathology in *Galc* SC cKO nerves. P60 sciatic nerve EM cross‐sections were analyzed for morphologically abnormal axons, defined by abnormal axoplasmic morphology with conspicuous intracellular organelle accumulation relative to normal axons. The abundance of morphologically abnormal axons was similar between *Galc* SC cKO and *Atg7‐Galc* SC cKO nerves (Figure 6C), indicating that *Atg7* deletion did not further increase this ultrastructural axonal phenotype at P60. Consistent with this, axon density was markedly reduced in both *Galc* SC cKO and *Atg7‐Galc* SC cKO sciatic nerves compared with WT, but was not further reduced by *Atg7* deletion in the *Galc* SC cKO background (Figure 6D). Lastly, we counted the number of membrane‐bound myelin‐containing structures in the SC cytoplasm. This revealed that deleting *Atg7* in the *Galc* SC cKO model tended to increase both the percentage of Schwann cells containing membrane‐bound myelin‐containing structures and the number of myelin‐containing structures per Schwann cell (Figure 6E). Taken together, these data indicate that, despite the additional reduction in myelin thickness, *Atg7* deletion did not further increase macrophage abundance or the prevalence of morphologically abnormal axons in *Galc* SC cKO nerves at P60.

### 3.5 Atg7 Deletion in KD SCs Reduces Rotarod Performance and Nerve Conduction Velocity

We next examined the physiological effects of *Atg7* deletion in WT and *Galc* SC cKO mice using rotarod motor coordination and nerve electrophysiology tests. Consistent with a previous report (Gomez‐Sanchez et al. 2015), the loss of *Atg7* alone in normal development did not affect rotarod performance at P35 or P60 (Figure 7A). In contrast, *Atg7‐Galc* SC cKO mice performed significantly worse than *Galc* SC cKO mice on the rotarod test at P60 where *Atg7‐Galc* SC cKO mice were unable to continue and consequently fell on the test floor after the first few seconds on the accelerating rotarod. Similarly, *Atg7‐Galc* SC cKO mice showed significantly impaired nerve conduction compared with *Galc* SC cKO mice at P60 (Figure 7B). We observed a decrease in nerve conduction velocity (NCV) as well as an increase in the proximal latency in *Atg7‐Galc* SC cKO mice. A decrease in NCV indicates slower electrical signal transmission due to factors like demyelination, axonal damage, nerve compression, inflammation, or metabolic/toxic effects (Preston and Shapiro 2013; Tavee 2019). Proximal latency refers to the time delay between the initiation of a stimulus and the onset of a measurable response near the point of stimulation and is particularly useful in diagnosing and monitoring peripheral neuropathies as a prolonged proximal latency can indicate nerve damage or dysfunction (Preston and Shapiro 2013; Tavee 2019). We monitored *Atg7‐Galc* SC cKO mice for up to six months and did not observe a significant difference in overall survival compared with *Galc* SC cKO mice. However, *Atg7‐Galc* SC cKO mice exhibited poorer rotarod performance and slightly worse hindlimb paresis. Collectively, these data show that *Atg7* deletion in *Galc* SC cKO mice resulted in worsening of rotarod and nerve electrophysiological performance, indicative of added nerve dysfunction.

**FIGURE 7:**
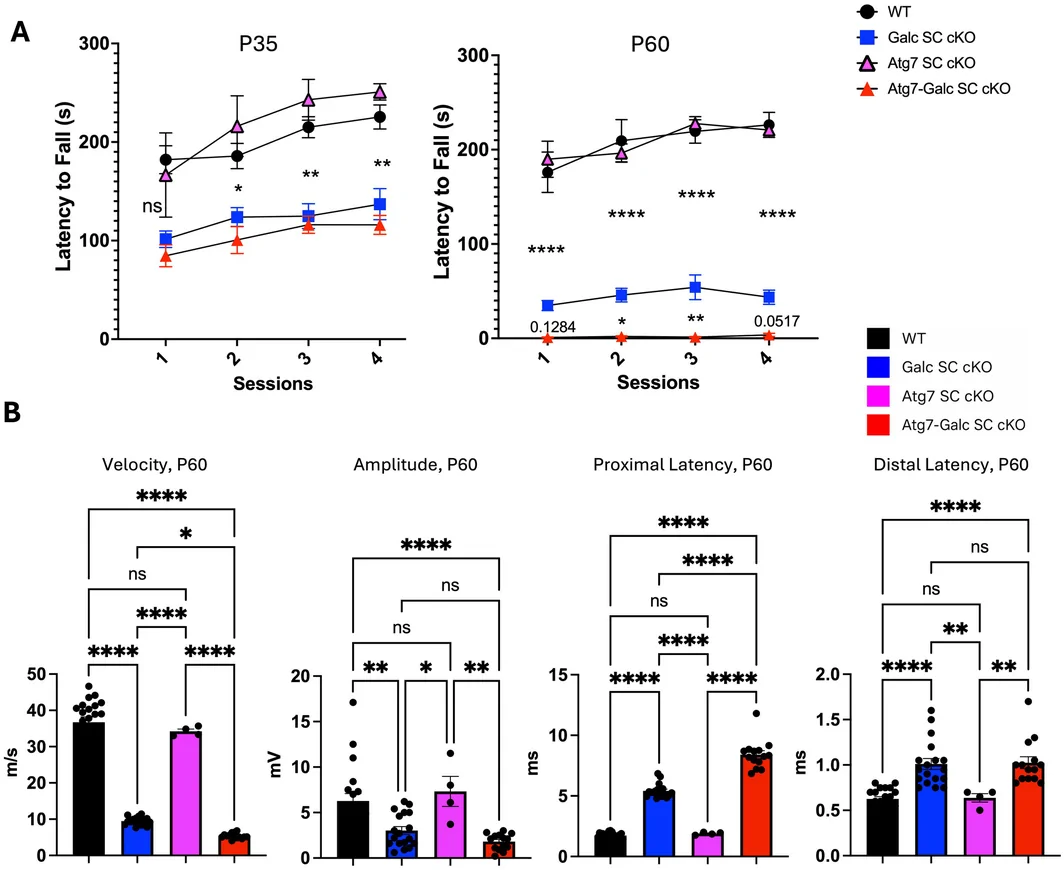
*Atg7* ablation in KD SCs worsens rotarod and electrophysiological performance at P60. A. Rotarod analysis of P35 and P60 WT, *Atg7* SC cKO, *Galc* SC cKO, and *Atg7‐Galc* SC cKO mice. Rotarod performance was measured across four test sessions. Sessions 1–4 were sequential trials, performed over the course of 2 days, in the morning and the evening. Each session value represents the average of three trials per animal. Each point represents the mean latency to fall for all animals within each genotype at the indicated session. Six to nine animals per genotype were analyzed. Groups were compared by two‐way ANOVA as a repeated‐measures analysis at each age. B. Sciatic nerve conduction analysis of conduction velocity, amplitude, and latency in P60 WT, *Atg7* SC cKO, *Galc* SC cKO, and *Atg7‐Galc* SC cKO mice. Bilateral sciatic nerve recordings were obtained from each animal, and measurements from the two nerves were averaged to generate a single animal‐level value for statistical analysis. Each dot represents one animal; at least four animals per genotype were analyzed. Groups were compared by one‐way ANOVA. *p < 0.05; **p < 0.01; ***p < 0.001; ****p < 0.0001; n.s. = not significant. Data are presented as mean ± SEM.

## 4 Discussion

KD pathology involves progressive demyelination, neuroinflammation, and neurological decline (LeVine and Torres 1992; Mikoshiba et al. 1985). Multiple therapeutic strategies such as hematopoietic stem cell transplantation, gene therapy, substrate reduction therapy, enzyme replacement therapy, and cell‐based approaches have been explored in the *twitcher* mouse, with variable success (Bradbury et al. 2020; Heller et al. 2023). Although recent preclinical and clinical gene therapy trials in large animal models have shown encouraging results (Bradbury et al. 2020; Heller et al. 2023), disease progression remains challenging to fully halt, underscoring the need to better define the cellular mechanisms driving pathology.

Autophagy has emerged as an important modifier of pathology in LSDs (Lieberman et al. 2012; Seranova et al. 2017). In LSDs where lysosomal enzymes are defective or insufficient (Vellodi 2005), autophagy assumes a compensatory role by facilitating the clearance of aggregated substrates and dysfunctional organelles (Lieberman et al. 2012; Seranova et al. 2017). Recent studies using rapamycin to enhance autophagy in *twitcher* reported reduced neuroinflammation and modest structural improvements, although functional gains were limited (Del Grosso et al. 2024; Lin et al. 2023). These studies have limitations. For example, the *twitcher* mouse remains a widely used model for infantile KD but does not fully represent later‐onset forms. Similarly, rapamycin, although frequently used to stimulate autophagy, acts primarily via mTOR inhibition and has broad effects beyond autophagy induction. Nevertheless, these findings highlight the potential, yet incomplete, benefits of autophagy modulation and emphasize the need to define how autophagy contributes to KD progression at the cell‐specific level.

In our SC‐specific GALC‐deficient model, we observed an early increase in the percentage of Schwann cells containing yellow LC3 reporter puncta beginning at P7, prior to overt myelin abnormalities. This suggests that changes in LC3 reporter‐positive structures occur early in response to GALC deficiency, although the direction of autophagic flux cannot be determined from the present analysis. These early autophagy‐associated changes align with previous findings in *twitcher*, where subtle PNS abnormalities precede demyelination (Suzuki 2003; Tanaka et al. 1988). Our results extend these observations by identifying Schwann cells as a key site of early autophagy‐associated changes in KD.

Unexpectedly, deleting *Atg7* in *Galc* SC cKO mice altered the composition of LC3 reporter‐positive puncta at P35. Although total red puncta numbers were not significantly different among genotypes, *Atg7‐Galc* SC cKO nerves had an increase in yellow puncta per field compared with *Galc* SC cKO nerves. The proportion of yellow puncta relative to total red puncta was also increased in *Atg7‐Galc* SC cKO nerves, indicating a shift toward the double‐positive LC3 reporter population rather than a generalized increase in total reporter puncta. The biological basis of this shift remains unresolved. Because ATG7 deficiency impairs canonical LC3 lipidation, the yellow puncta observed in ATG7‐deficient Schwann cells cannot be assumed to represent canonical autophagosomes and may reflect non‐lipidated reporter aggregation, altered LC3 reporter handling, or potentially ATG7‐independent pathways, such as RAB9‐dependent autophagy, described under conditions of cellular stress (Arakawa et al. 2017; Nishida et al. 2009). Also, we cannot exclude the possibility of residual ATG7 activity after Cre‐mediated deletion in Schwann cells (Figure 3A). Residual ATG7 expression or incomplete recombination may provide sufficient autophagic capacity under basal conditions but become inadequate when cellular demand is increased by GALC deficiency and associated lysosomal and myelin stress. In this setting, reduced autophagic reserve could contribute to the accumulation of altered LC3 reporter‐positive structures and exacerbate myelin pathology. However, the extent of *Atg7* recombination and residual ATG7 activity at the single‐cell level was not directly assessed, and this possibility therefore remains speculative. Importantly, these reporter measurements do not establish increased autophagosome formation, impaired autophagosome‐lysosome fusion, or the direction of autophagic flux. Future studies incorporating direct turnover assays, lysosomal inhibition, and complementary lysosomal function measurements will be necessary to determine the mechanisms underlying these altered LC3 reporter‐positive structures. Although *Atg7* deletion did not disrupt developmental myelination, its loss in the context of GALC deficiency resulted in thinner myelin at P60 and impaired motor and electrophysiological performance. While the electrophysiological abnormalities observed in *Atg7‐Galc* SC cKO mice are consistent with impaired peripheral nerve conduction, nodal and paranodal architectures were not directly assessed in this study. Therefore, these functional abnormalities should not be interpreted as direct evidence of structural disruption of the Node of Ranvier. These findings indicate that ATG7‐dependent pathways support myelin maintenance during disease progression, even if they are not required for initial developmental myelination. The selective exacerbation of myelin thinning without an accompanying increase in macrophage abundance suggests that loss of ATG7‐dependent pathways worsens myelin integrity without further increasing macrophage recruitment at this stage.

An additional mechanism that may contribute to the worsened myelin phenotype following *Atg7* deletion is altered handling of destabilized myelin membranes. Psychosine accumulation has been shown to increase local membrane rigidity and promote microvesiculation and shedding of myelin membranes, providing a potential mechanism by which psychosine directly contributes to myelin destabilization in KD (D'Auria et al. 2017). In parallel, recent work in peripheral nerve injury demonstrated that demyelinating Schwann cells can reprogram ATG7‐dependent autophagy toward a secretory pathway, in which ATG7‐dependent secretory phagophore formation contributes to the exocytosis of myelin material for subsequent extracellular disposal (Jo et al. 2023). In the context of GALC deficiency, psychosine‐mediated membrane destabilization could therefore increase the burden of fragmented or damaged myelin requiring intracellular and extracellular disposal, while loss of ATG7 may compromise one component of this adaptive myelin‐handling response. Such a mechanism could contribute to the exacerbated myelin thinning observed in *Atg7‐Galc* SC cKO nerves. However, myelin membrane shedding, secretory autophagy, and subsequent extracellular clearance were not directly examined in the present study; therefore, this model remains an alternative hypothesis that will require direct experimental investigation.

Together, our findings support a model in which GALC deficiency is associated with early changes in autophagy‐related proteins and LC3 reporter‐positive structures in Schwann cells. Loss of ATG7‐dependent autophagy selectively worsens myelin maintenance and functional outcomes without increasing macrophage abundance or the prevalence of morphologically abnormal axons at P60, supporting a protective role for ATG7‐dependent canonical autophagy in myelin maintenance during disease progression. These findings identify ATG7‐dependent Schwann‐cell pathways as potential modifiers of myelin maintenance. Additional studies are required to determine whether therapeutic modulation of autophagy would be beneficial in GALC‐deficient nerves.

### 4.1 Limitations

Several limitations of this study should be considered. First, although we used a Schwann cell‐specific GALC knockout model to isolate the peripheral contribution of lysosomal dysfunction, this approach does not capture the full multisystem complexity of KD, in which central nervous system pathology, immune interactions, and metabolic disturbances also play major roles. Second, the *Atg7* deletion specifically interrogates canonical autophagy; however, ATG7‐independent pathways such as RAB9‐mediated alternative autophagy may compensate, potentially confounding interpretation of autophagic flux and limiting our ability to attribute phenotypes solely to loss of canonical autophagy. Third, LC3 and p62 were measured under steady‐state conditions without lysosomal inhibition or turnover analysis. Therefore, the present study does not determine the direction or magnitude of autophagic flux. Fourth, our tandem LC3 reporter analysis quantified total red, yellow, red only, and the relative proportion of yellow puncta at the animal level. Because individual Schwann cell boundaries could not be reliably delineated across all analyzed images, puncta counts were normalized to imaging field number rather than Schwann cell number. Moreover, the reporter measurements do not directly determine autophagic flux or lysosomal degradative capacity in vivo. Complementary assays such as tandem LC3 turnover or lysosomal pH measurements would provide greater mechanistic resolution. Furthermore, the identity of the reporter‐positive puncta in *Atg7*‐deficient Schwann cells was not independently validated, and these structures cannot be assumed to represent canonical autophagosomes. Additionally, although we assessed macrophage abundance and ultrastructural axonal morphology, functional characterization of immune phenotypes, cytokine signaling, complementary molecular markers of axonal injury, and axon‐glia metabolic coupling was beyond the scope of this study. Finally, the study design concentrated on disease progression up to P60; longer‐term analyses and therapeutic perturbations will be necessary to determine whether manipulating Schwann cell autophagy can meaningfully alter clinical outcomes beyond myelin maintenance. Together, these limitations highlight the need for expanded mechanistic and longitudinal studies to fully define the therapeutic potential of autophagy modulation in KD.

### 4.2 Future Directions

Future studies should explore whether selectively modulating ATG7‐dependent autophagy‐related pathways can improve myelin stability in GALC‐deficient nerves, particularly through approaches that avoid broad mTOR inhibition. Defining how GALC deficiency shifts cells toward ATG7‐independent autophagy, and whether reinforcing or modulating these alternative pathways can compensate for disruption of ATG7‐dependent canonical autophagy, will be important. Additionally, integrating metabolic profiling, lysosomal function assays, and live imaging of autophagosome‐lysosome fusion could help determine the direction of autophagic flux and clarify the mechanisms underlying altered LC3 reporter‐positive structures. Finally, extending analyses beyond P60 and combining autophagy‐targeted strategies with emerging gene or enzyme replacement therapies may reveal synergistic therapeutic windows capable of stabilizing or improving peripheral nerve function in KD.

## Author Contributions

Conceptualization by N.D., M.L.F. Animal handling, tissue collection and preparation, western blotting, immunohistochemistry experiments conducted by N.D., A.S., J.Z., T.R. Semithin and EM experiments conducted by E.H., T.R. Rotarod and electrophysiology studies conducted by N.D. Original draft written by N.D. Manuscript reviewed and edited by N.D., P.M.P., M.L.F., F.J.S, D.S. Supervised by D.S, M.L.F. All authors have read and agreed to the published version of the manuscript.

## Funding

This work was supported by grants from National Institutes of Health‐R01NS111715 (to M.L.F.), R01NS112327 (to D.S.), F31NS124106 (to N.D.), and Rosenau Family Research Foundation (to M.L.F.).

## Ethics Statement

The animal study protocols (UB1188 and UB1254M) were approved by the Institutional Animal Care and Use Committee (IACUC) of the University at Buffalo, the State University of New York.

## Conflicts of Interest

The authors declare no conflicts of interest.

## Supporting information


**Supplemental Figure 1.** Autophagy and lysosomal protein expression in P60 sciatic nerves. Western blot analysis of p62, LC3, and LAMP1 in P60 sciatic nerves from WT, Galc SC cKO, Atg7 SC cKO, and Atg7‐Galc SC cKO mice. GAPDH was used as a loading control (related to Figure 5).

**Supplemental Figure 2.** RFP+/EGFP+ (yellow) signals were classified as double‐positive LC3 reporter puncta, whereas RFP+ only signals were classified as red LC3 puncta. Quantification shows total red LC3 reporter puncta per field, yellow LC3 reporter puncta per field, red only LC3 reporter puncta per field, and the percentage of yellow puncta relative to total red puncta. Puncta counts were normalized to the number of fields analyzed for each animal. WT, *Galc* SC cKO, and *Atg7‐Galc* SC cKO groups contained four animals each, and the *Atg7* SC cKO group contained three animals. Scale bar = 10 μm. Each dot represents one animal. Ordinary one‐way ANOVA followed by Tukey's multiple‐comparisons test was performed. *p < 0.05; **p < 0.01; ***p < 0.001 (related to Figure 6).

**Supplemental Figure 3.** Full, uncropped western blot images corresponding to Figures 2A, 3A, and 5. Highlighted regions correspond to bands presented in the main figures (related to all WB analyses).

## Data Availability

Further information and requests for resources and reagents should be directed to and will be fulfilled by the Lead Contact, Daesung Shin (daesungs@buffalo.edu), and the corresponding author, Narayan Dhimal (narayand@buffalo.edu). There are no restrictions on any data or materials presented in this paper.
